# Clinicopathological and molecular characteristics of *RSPO* fusion-positive colorectal cancer

**DOI:** 10.1038/s41416-022-01880-w

**Published:** 2022-06-17

**Authors:** Taiki Hashimoto, Daisuke Takayanagi, Junpei Yonemaru, Tomoaki Naka, Kengo Nagashima, Yasushi Yatabe, Dai Shida, Ryuji Hamamoto, Sam O Kleeman, Simon J Leedham, Atsuo Takashima, Kouya Shiraishi, Shigeki Sekine

**Affiliations:** 1 Division of Diagnostic Pathology, National Cancer Center Hospital, Tokyo, Japan; 2 Division of Genome Biology, National Cancer Center Research Institute, Tokyo, Japan; 3 Biostatistics Unit, Clinical and Translational Research Center, Keio University Hospital, Tokyo, Japan; 4 Division of Molecular Pathology, National Cancer Center Research Institute, Tokyo, Japan; 5 Division of Colorectal Surgery, National Cancer Center Hospital, Tokyo, Japan; 6 Division of Frontier Surgery, The Institute of Medical Science, Tokyo, Japan; 7 Division of Medical AI Research and Development, National Cancer Center Research Institute, Tokyo, Japan; 8 Intestinal Stem Cell Biology Lab, Welcome Trust Centre Human Genetics, University of Oxford, Oxford, UK; 9 Division of Gastrointestinal Medical Oncology, National Cancer Center Hospital, Tokyo, Japan

**Keywords:** colorectal cancer, *RSPO* fusion

## Abstract

**Background:**

*RSPO* fusions that lead to WNT pathway activation are potential therapeutic targets in colorectal cancer (CRC), but their clinicopathological significance remains unclear.

**Methods:**

We screened 1019 CRCs for *RSPO* fusions using multiplex reverse transcription–PCR. The *RSPO* fusion-positive tumors were subjected to whole-exome sequencing (WES).

**Results:**

Our analysis identified 29 CRCs with *RSPO* fusions (2.8%), consisting of five with an *EIF3E-RSPO2* fusion and 24 with *PTPRK-RSPO3* fusions. The patients were 17 women and 12 men. Thirteen tumors (45%) were right-sided. Histologically, approximately half of the tumors (13/29, 45%) had a focal or extensive mucinous component that was significantly more frequent than the *RSPO* fusion-negative tumors (13%; p = 8.1 × 10^−7^). Four tumors (14%) were mismatch repair-deficient. WES identified *KRAS, BRAF*, and *NRAS* mutations in a total of 27 tumors (93%). In contrast, pathogenic mutations in major WNT pathway genes, such as *APC, CTNNB1*, and *RNF43*, were absent. *RSPO* fusion status did not have any statistically significant influence on the overall or recurrence-free survival. These clinicopathological and genetic features were also confirmed in a pooled analysis with previous studies.

**Conclusion:**

*RSPO* fusion-positive CRCs constitute a rare subgroup of CRCs with several characteristic clinicopathological and genetic features.

## Background

Recent large-scale sequencing projects have revealed the mutational landscape of colorectal cancer (CRC) and indicated that virtually all CRCs have genetic alterations related to the WNT signaling pathway [1–4]. These observations suggest that constitutive activation of the WNT signaling pathway plays an essential role in the development of CRC. In particular, approximately 80% of CRCs harbor loss-of-function mutations of *APC* or activating mutations of *CTNNB1*, both of which lead to cell autonomous activation of the WNT pathway [4].

Recently, *EIF3E-RSPO2* and *PTPRK-RSPO3* fusions were identified as recurrent genetic alterations in CRC [5]. These fusions consist of the 3′ region of *RSPO* and the 5′ region of *EIF3E* or *PTPRK*, resulting in the overexpression of functional R-spondin proteins. R-spondins potentiate WNT signaling by downregulating the E3 ubiquitin ligases RNF43 and ZNRF3, which promote the endocytosis of WNT receptors [6]. Because *RSPO* fusions were detected in CRCs lacking *APC* and *CTNNB1* mutations, they are thought to be alternative mechanisms to induce WNT pathway activation [5].

Although activation of the WNT pathway is a ubiquitous molecular abnormality in CRCs, there have been no molecular therapies targeting this pathway to date because of the lack of appropriate inhibitors for clinical use. However, recent studies have implied that *RSPO* fusion-positive CRCs might be excellent candidates for WNT-targeted therapies because of their distinct mode of WNT activation. In contrast to other WNT-related genetic alterations, *RSPO* fusion products are secreted proteins and therefore targetable by antibodies [7, 8]. Alternatively, because *RSPO* fusions enhance the ligand-dependent activation of the WNT signaling pathway, inhibition of WNT ligand secretion by PORCN inhibitors is also expected to be effective [9–11]. In fact, several in vitro and patient-derived xenograft models have demonstrated the efficacy of these strategies in the treatment of tumors with an *RSPO* fusion [7–11].

Despite their potential clinical significance, only a limited number of CRCs harboring *RSPO* fusions have been described [5, 11–17]. Moreover, as many of previous reports lacked clinical data, there is no clear information on the clinicopathological features of *RSPO* fusion-positive CRCs. Therefore, to better characterize CRCs with an *RSPO* fusion, we screened a large cohort of CRCs for previously reported recurrent *RSPO* fusions and investigated their clinical, histological, and molecular features.

## Methods

### Patient selection

We analyzed 1019 primary CRC cases surgically treated at the National Cancer Center Hospital, Tokyo, Japan, between 1997 and 2019. Cases available for frozen tissue materials were included in this study. We retrieved details regarding the age, sex, tumor location, histological diagnosis, tumor grade, pathological stage, and outcome of each patient. Staging was based on the UICC TNM classification (8th edition). Left-sided tumors included tumors of the left colon and the rectum.

### Multiplex reverse transcription–polymerase chain reaction

RNA and DNA samples were extracted from fresh-frozen tissue materials using AllPrep Kit (QIAGEN, Valencia, CA). Multiplex reverse transcription–polymerase chain reaction (RT-PCR) targeting all previously reported recurrent *RSPO* fusions, an *EIF3E-RSPO2* fusion and four *PTPRK-RSPO3* fusion variants [5, 18, 19], was conducted using the primers listed in Supplementary Table 1. The PCR products were subjected to Sanger sequencing to identify specific fusion transcripts.

### Histological analysis

Histological diagnosis was made according to the World Health Organization classification [20]. Tumors were classified into low-grade (well and moderately differentiated) or high-grade (poorly differentiated) based on the predominant histology. For *RSPO* fusion-positive tumors, we reviewed all of the available tumor slides and evaluated the following histological findings: lymphovascular invasion, perineural invasion, mucinous component, tumor infiltrating lymphocytes (TILs), Crohn’s-like reaction, and association of a precursor component. The areas of extracellular mucin were semiquantitatively evaluated as < 10%, 10–50%, or > 50% of the tumor area. Tumor infiltrating lymphocytes were determined as the average number of lymphocytes within the tumor epithelium per high-power field (HPF) by counting five HPFs. Tumors with ≥ 2 TILs per HPF were classified as high-level TILs [21]. Crohn’s-like reaction was considered to be present if three or more lymphoid aggregates were detected per section [21]. For comparison, *RSPO* fusion-negative tumors were also histologically reviewed for the presence of mucinous components.

### Whole-exome sequencing

Whole-exome sequencing (WES) was conducted using 200 ng of genomic DNA isolated from cancerous and non-cancerous tissue samples obtained from all *RSPO* fusion-positive cases. Exome capture was performed using the SureSelect Human All Exon V6 (Agilent Technologies, Santa Clara, CA, USA) according to the manufacturer’s instructions. Exome sequencing was performed on the Illumina NovaSeq 6000 platform using 2 × 150 bp paired-end reads (Illumina, San Diego, CA, USA). Basic alignment and sequence quality control were conducted according to the GATK4 best practices pipeline [22, 23]. The reads were aligned against the reference human genome from the UCSC human genome 19 (hg19). Somatic single nucleotide variants and insertion/deletion mutations were called using the Mutect2 program (ver 4.1.2.0) [22]. Some commonly mutated genes were manually inspected using Integrative Genomics Viewer [24]. Variants annotated as oncogenic or likely oncogenic in OncoKB (https://www.oncokb.org/) were considered to be pathogenic.

### Immunohistochemistry and methylation-specific PCR

Immunohistochemistry for four mismatch repair (MMR) proteins, MLH1, MSH2, PMS2, and MSH6, was performed on a tissue microarray and/or whole tissue sections as previously described [25]. Tumors showing loss of any MMR protein expression were considered as MMR-deficient, and those retaining all the four MMR proteins were considered as MMR-proficient. The tumors that showed loss of MLH1 expression were subjected to an *MLH1* promoter methylation analysis. Methylation-specific PCR of the *MLH1* promoter was conducted as described previously [26].

### Review of the previously reported *RSPO* fusion-positive CRCs

To test the consistency of our observations, we reviewed clinicopathological and molecular features and prognosis of *RSPO* fusion-positive CRCs in previously reported large-scale studies: TCGA [1], S:CORT [15], and Genentech cohorts [5]. The data were retrieved from the respective papers except that clinicopathological and prognostic data of TCGA cohort was downloaded from the Broad GDAC Firehose (https://gdac.broadinstitute.org/) and cBioPortal (https://www.cbioportal.org), respectively. In the TCGA cohort, the areas of extracellular mucin were determined by reviewing histological images available at cBioportal. Mutation profiles of the TCGA cohort were adapted from the study by Grasso et al [27].

### Statistical analysis

Chi-square test and Welch’s t test were used to analyze categorical and continuous variables, respectively. Univariate and multivariate logistic regression models were used to analyze the predictors for the presence of *RSPO* fusion. Overall survival (OS) and recurrence-free survival (RFS) of patients with stage II and III CRC who underwent tumor-free resection were estimated using the Kaplan–Meier method, and the survival difference was compared using the log-rank test. OS and RFS were defined as the time from the date of surgery to the date of death and recurrence, respectively. We also applied Cox regression models to evaluate the prognostic significance of the individual variables. Two-sided P values < 0.05 were considered to indicate statistical significance. SAS version 9.4 (SAS Institute, Cary, NC, USA) was used for statistical analyses.

## Results

### Detection of *RSPO* fusion transcripts

The multiplex RT-PCR-based screening of 1019 CRCs identified *RSPO* fusions in 29 tumors (2.8%). *EIF3E-RSPO2* and *PTPRK-RSPO3* fusion transcripts were detected in 5 and 24 tumors, respectively. The most frequent *PTPRK-RSPO3* fusion variant was *PTPRK* exon *1-RSPO3* exon 2 (18 cases), followed by *PTPRK* exon 7-*RSPO3* exon 2 (4 cases), and *PTPRK* exon 13-*RSPO3* exon 2 (2 cases).

### Clinicopathological features

**Figure 1:**
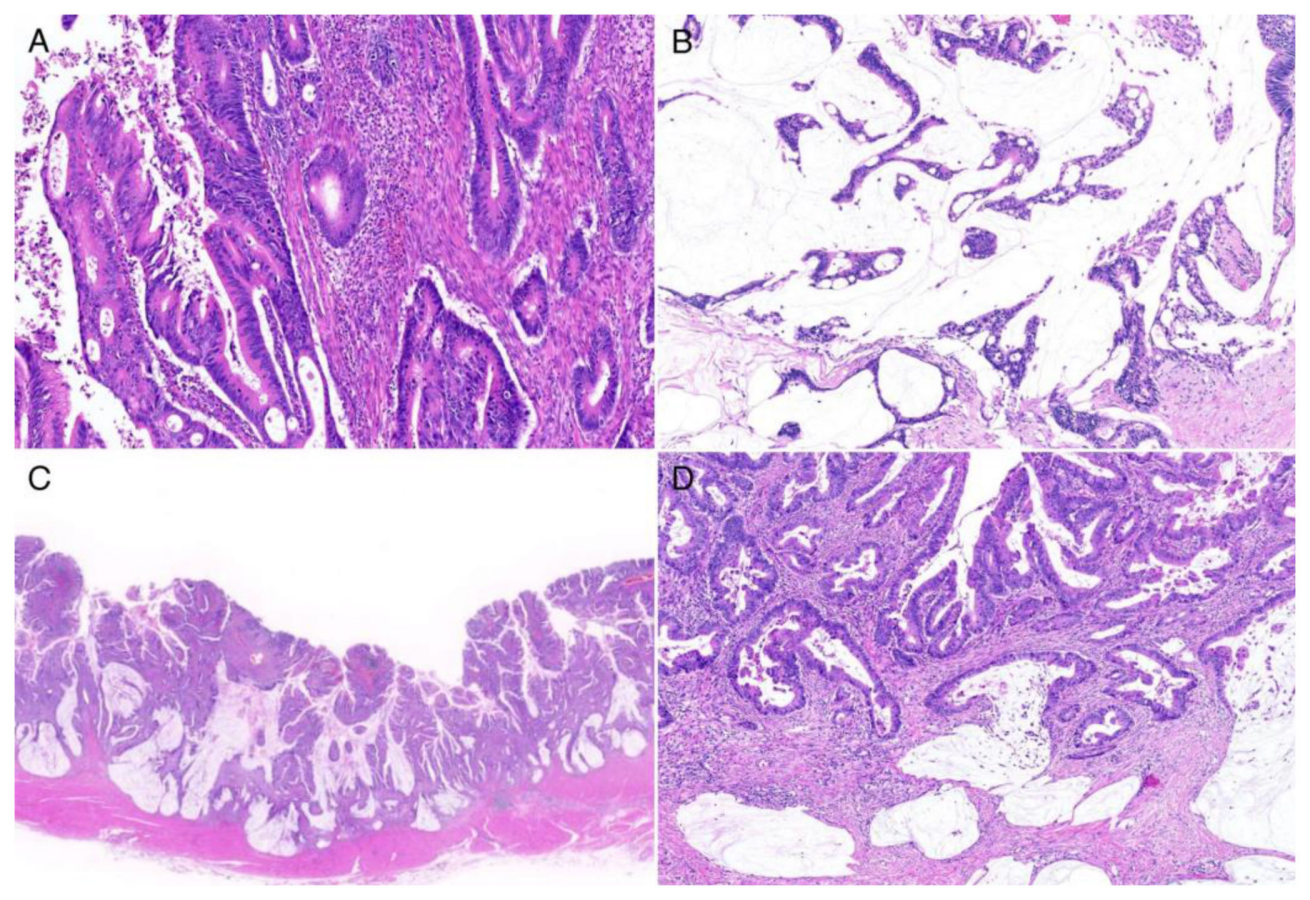
Histology of *RSPO* fusion-positive colorectal cancer. **A:** Tubular adenocarcinoma, not otherwise specified. **B:** Mucinous carcinoma exhibiting extensive extracellular mucin and gland-forming tumor cells. **C, D:** Adenocarcinoma with a focal mucinous component. The mucinous component occupies less than 50% of the tumor area (C). Mucin production was mainly observed at the invasive edge of the tumor (D).

*RSPO* fusion-positive CRCs were detected in 17 women and 12 men whose median age was 64 years (Table 1). Thirteen tumors were located in the right colon, 8 were in the left colon, and 8 were in the rectum (Supplementary Table 2). Histologically, the majority of CRCs with *RSPO* fusions were adenocarcinomas, not otherwise specified (24/29, 83%; Figure 1A and Supplementary Table 3). Four cases were mucinous adenocarcinomas (Figure 1B). Focal mucinous components, constituting 10–50% of the tumor area, were observed in nine tumors and were mostly located at the invasive edges (Figure 1C, D). Collectively, nearly half of the tumors (13/29, 45%) had a focal or extensive mucinous component. One tumor was medullary carcinoma. Lymphatic and venous invasion was detected in 15 and 14 tumors, respectively. Eight tumors showed high-level TILs. Crohn’s-like reaction was present in 12 tumors. A precursor component was detected in two tumors, one case with a tubulovillous adenoma and another case with a villous adenoma. The prevalence of lymphatic and vascular invasion, high level TILS, and Crohn’s-like reaction was similar to those of CRCs reported in previous studies [21, 28, 29], but tumors with a mucinous component in *RSPO* fusion-positive tumors was much more frequent compared with that reported previously [30, 31]. Therefore, we additionally reviewed 990 *RSPO* fusion-negative CRCs for comparison and identified 127 tumors (13%) with a focal or extensive mucinous component.

Compared with CRCs without *RSPO* fusions, *RSPO* fusion-positive tumors were more likely to occur in women (p = 0.036), to be located in the right colon (p = 0.044), and to have a mucinous component (p = 8.1 × 10^−7^). The univariate analysis showed that female gender (odds ratio [OR], 2.2; 95% confidence interval [CI], 1.0–4.5), right-sided location (OR, 2.1; 95% CI, 1.0–4.4), and the presence of mucinous component (OR, 5.5; 95% CI, 2.6–12) were associated with the presence of an *RSPO* fusion (Table 2). In the multivariate analysis, we observed that female gender (OR, 2.2; 95% CI, 1.0–4.8) and the presence of a mucinous component (OR, 5.3; 95% CI, 2.5–11) were independently associated with *RSPO* fusions.

Previous reports were reviewed to explore if these clinicopathological characteristics of *RSPO* fusion-positive CRCs are common to other cohorts. The pooled results of the present and four previously reported studies also showed a significant association of *RSPO* fusions with the female gender (p = 0.033; Supplementary Table 4) and the presence of mucinous components (p = 6.9 × 10^−8^). In contrast, the preferential localization to the right colon was not reproduced in the pooled analysis (p = 0.10).

### Mutation profiles of *RSPO* fusion-positive CRCs

The tumor and matched normal tissue samples of all *RSPO* fusion-positive cases were subjected to WES. Mean sequencing depth was 149.6× (89.6–216.1×) for tumor samples and 105.0× (70.2–153.8×) for matched normal samples. On average, 93.1% of bases were covered by at least 20 reads in each sample. A total of 29317 somatic mutations, including 6788 missense, 80 inframe insertion and deletion, 361 nonsense, 1071 frameshift, 1017 splicing and 2794 silent mutations, were identified. The median tumor mutational burden (TMB) was 3.9 mutations per megabase (Mb), and 4 cases had a high TMB (> 10).

**Figure 2:**
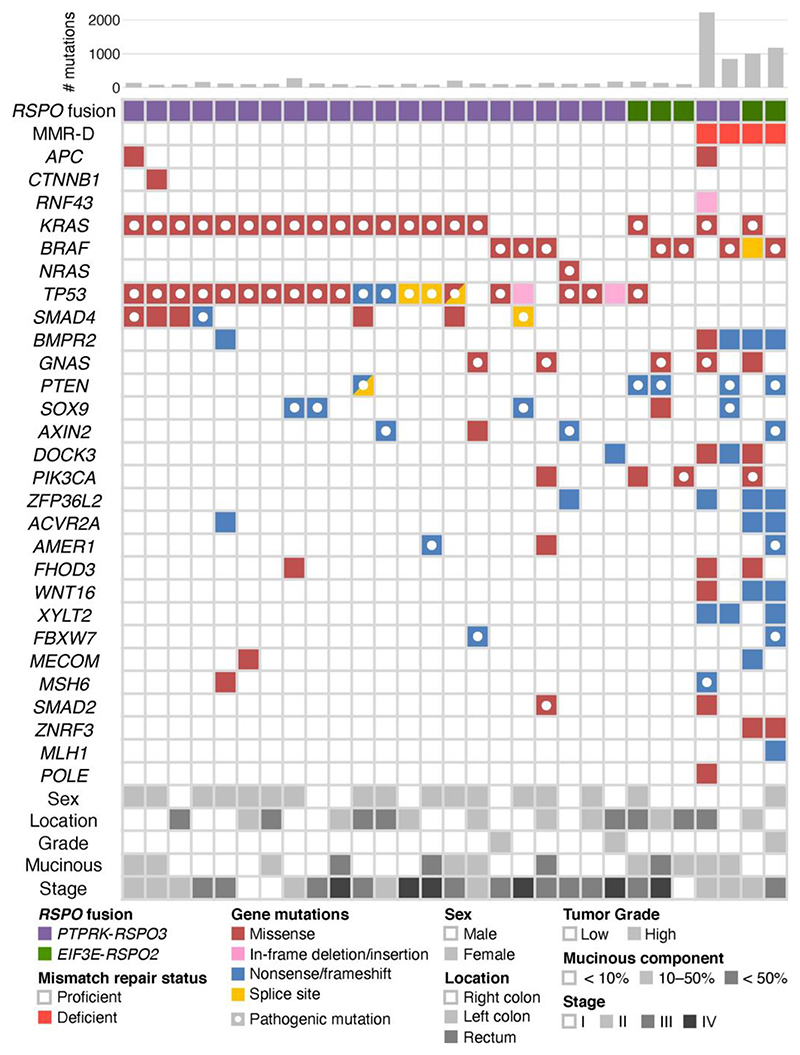
Summary of whole-exome sequencing analysis. Genes recurrently mutated in colorectal cancers, based on previous studies [2], are presented. Open circles indicate pathogenic mutations.

Three tumors had WNT pathway-related gene mutations, including *APC*, *RNF43*, and *CTNNB1* (Figure 2), but all of these were regarded as nonpathogenic following the OncoKB annotation. Activating mutations of *KRAS, BRAF*, and *NRAS* were detected in 19, 7, and 1 tumor, respectively. These *RAS/RAF* mutations were mutually exclusive; therefore, all but two *RSPO* fusion-positive CRCs harbored one of these MAPK pathway mutations (27/29, 93%). Pathogenic *TP53* mutations were detected in 16 tumors (55%). Two tumors had somatic MMR gene mutations, a frameshift mutation of *MLH1* and a nonsense mutation of *MSH6*. Among the other common genetic alterations in CRC, pathogenic *PTEN*, *GNAS*, *SMAD4*, and *PIK3CA* mutations were present in 5, 4, 3, and 2 tumors, respectively.

Next, previously reported mutation data on *RSPO* fusion-positive CRCs were reviewed. The results verified the almost complete absence of pathogenic mutations in WNT pathway-related genes and highly frequent MAPK gene mutations (Table 3). Among a total of 55 *RSPO* fusion-positive CRCs, including those analyzed in the present study, only one tumor had a truncating *APC* mutation (2%), whereas *RNF43* and *CTNNB1* mutations were absent. *KRAS*, *BRAF*, and *NRAS* mutations were detected in 33 (62%), 15 (27%), and 2 cases (4%), and collectively, 50 cases (91%) had one of these MAPK pathway gene mutations.

### Expression of MMR proteins

Immunohistochemistry for MMR proteins was performed on 1014 tumors. Loss of any MMR protein expression, indicative of MMR deficiency, was observed in 4 tumors with *RSPO* fusions (14%) and 85 tumors without *RSPO* fusions (9%). The prevalence of MMR-deficient tumors was not significantly different between tumors with and without *RSPO* fusions (Table 1). Among the four MMR-deficient CRCs with an *RSPO* fusion, three tumors showed concurrent MLH1 and PMS2 loss (Supplementary Figure 1), all of which were associated with *MLH1* promoter methylation (Supplementary Figure 2). One of the tumors with *MLH1* promoter methylation also had an *MLH1* mutation. Loss of MSH6 was observed in the tumor with an *MSH6* mutation. All of the MMR-deficient tumors exhibited a high TMB.

### Prognosis

**Figure 3:**
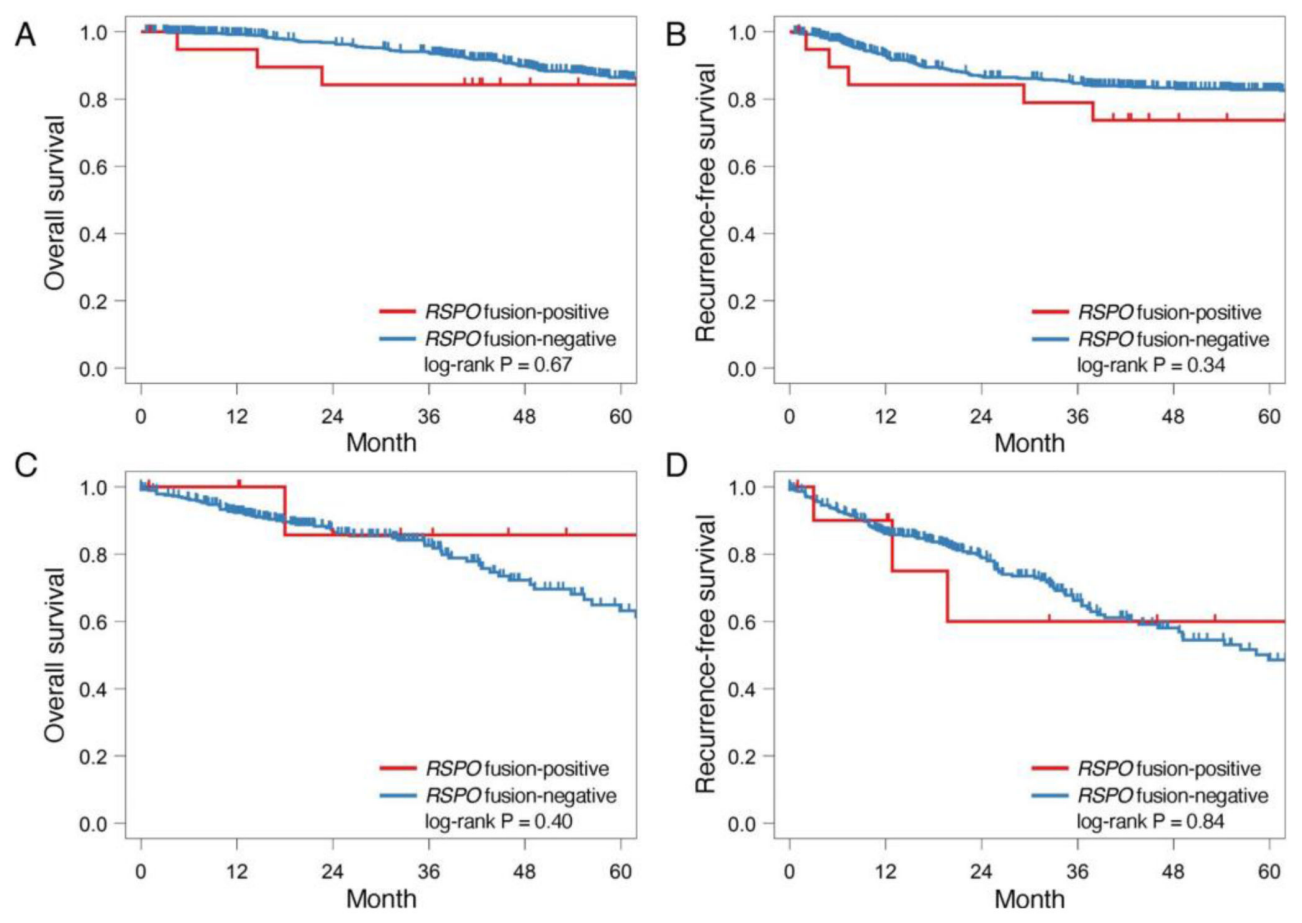
Kaplan–Meier curves, stratified by *RSPO* fusion status. Overall (A) and recurrence-free (B) survivals in all patients with stage II and III colorectal cancer in the present study cohort. Overall (C) and recurrence-free (D) survivals in all patients with stage II and III colorectal cancer in the TCGA cohort.

We investigated the prognostic role of *RSPO* fusions in 705 patients with stage II and III CRC who underwent complete resection, including 20 *RSPO* fusion-positive cases. The median follow-up after surgery was 1700 days (range, 20–7831 days). The presence of *RSPO* fusions was associated with slightly lower five-year OS and RFS rates, but these associations were not statistically significant (Figure 3A, B). We also performed a prognostic analysis of the TCGA cohort and did not find significant differences in OS and RFS between the *RSPO* fusion-positive and the fusion-negative groups (Figure 3C, D).

A univariate Cox regression analysis indicated that the presence of *RSPO* fusions tended to show a worse prognosis for OS (hazard ratio [HR], 1.3; 95% CI, 0.4–4.0; p = 0.69) and RFS (HR, 1.5; 95% CI, 0.63–3.8; p = 0.34); however, these trends were not statistically significant (Supplementary Table 4). In the multivariate analysis, older age, male gender, and higher stage were negatively correlated with OS, and male gender, left-sided location, and higher stage were correlated with shorter RFS. The presence of an *RSPO* fusion was associated with shorter OS and RFS (HR, 1.2; 95% CI, 0.39–4.0; p = 0.72, and HR, 2.3; 95% CI, 0.92–5.9; p = 0.073, respectively), but these associations were not statistically significant.

## Discussion

In this study, we characterized the clinicopathological and molecular features of the largest series of *RSPO* fusion-positive CRCs to date. Our RT-PCR-based screening detected *RSPO* fusions in 2.8% of CRCs, indicating their rarity among CRCs. Although the initial study conducted by Seshagiri et al reported a prevalence of 10.3% through the analysis of 68 CRCs [5], subsequent studies have reported a considerably lower prevalence of *RSPO* fusions (0.35–4%), similar to our observation [11-13, 15, 17]. Considering the relatively limited number of cases analyzed in the initial report, *RSPO* fusion-positive tumors appears to constitute a rare subgroup of CRCs.

This study revealed that *RSPO* fusion-positive CRCs have several clinicopathological features, including female predominance and the preferential localization to the right colon. The female predominance of *RSPO* fusion-positive CRCs was also verified in the pooled analysis of the present and previous studies. Furthermore, the presence of a mucinous component was significantly associated with *RSPO* fusions. Mucinous differentiation has been linked to several molecular features, including MMR deficiency [32, 33], *BRAF* mutation [34, 35], and *GNAS* mutation [36]. Although these molecular features can be concurrent with an *RSPO* fusion, 8 of the 13 *RSPO* fusion-positive CRCs with a mucinous component were MMR-proficient and lacked *BRAF* and *GNAS* mutations. Thus, our observation suggests that the *RSPO* fusion is another molecular feature associated with mucinous differentiation. In agreement with this notion, a recent study reported that CRCs with genetic abnormalities leading to ligand-dependent WNT activation, including *RSPO* fusions and *RNF43* mutations, have increased areas of mucus [15]. Notably, the diagnosis of mucinous adenocarcinoma based on the WHO classification requires a mucinous component occupying > 50% of the tumor volume; however, the present and previous studies have indicated that the presence of a minor mucinous component is sufficiently predictive of the molecular features associated with mucinous differentiation [31, 33, 37].

Consistent with the role of *RSPO* fusions in WNT pathway activation, the present and previous studies showed virtually complete absence of pathogenic *APC*, *CTNNB1*, and *RNF43* mutations in CRCs with an *RSPO* fusion. This observation supports an analogous role of *RSPO* fusions and *APC* mutations in activating the WNT pathway in colorectal tumorigenesis as previously suggested [5].

*KRAS*, *NRAS*, or *BRAF* mutations were detected in 27 of the 29 tumors in the present study. The high prevalence of MAPK pathway gene alteration was also shown in the pooled analysis with other cohorts. These observations may be attributable to the histogenesis of *RSPO* fusion-positive CRCs. Among colorectal polyps, *RSPO* fusions were exclusively detected in traditional serrated adenomas [38, 39]. Considering the almost consistent presence of *RAS*/*RAF* mutations in serrated polyps, it is reasonable to assume the specific role of *RSPO* fusions in the serrated pathway of tumorigenesis. However, unexpectedly, we found two *RSPO* fusion-positive CRCs with a conventional adenoma component. Thus, although previously unrecognized, *RSPO* fusions may be present in a minor subset of conventional-type adenomas.

In this study, four *RSPO* fusion-positive CRCs (14%) were found to be MMR-deficient. All the MMR-deficient tumors were unrelated to Lynch syndrome, with three cases exhibiting *MLH1* promoter methylation and one case exhibiting a somatic *MSH6* mutation. Although previous studies have described only isolated cases of microsatellite unstable CRCs with an *RSPO* fusion [11, 15], the prevalence of MMR deficiency was not significantly different between tumors with and without *RSPO* fusions in the present study. The recurrent association of MMR deficiency indicates the molecular heterogeneity of *RSPO* fusion-positive CRCs.

It has been reported that wild-type *APC* predicts a poor prognosis in CRC [40, 41]. Since *RSPO* fusion-positive CRCs lack *APC* mutations, we expected that they constitute the clinically aggressive subgroup of CRCs. However, prognostic analyses of the present study and TCGA cohorts did not support our expectation. These results suggest that *RSPO* fusion does not have a significant prognostic value and that a clinically aggressive subgroup may exist among *APC* wild-type and *RSPO* fusion-negative population.

A limitation of this study is that the cases included were selected based on the availability of frozen tissue samples and thus not a consecutive series. Moreover, because our screening targeted recurrent fusions, cases with rare types of *RSPO* fusions might have been missed. However, non-recurrent *RSPO* fusions are exceedingly rare according to the previous studies [5, 11, 13, 15, 17].

In summary, the present study illustrated the comprehensive clinicopathological and molecular characteristics of CRCs with an *RSPO* fusion. *RSPO* fusion-positive CRCs, constituting approximately 3% of CRCs, tended to occur in female patients and to be located in the right colon. The presence of focal or extensive mucinous components was a common histological feature. We confirmed that *RSPO* fusions are mutually exclusive with other WNT pathway gene mutations. This observation supports that targeting *RSPO* fusions is a valid therapeutic strategy for CRC.

## Supplementary Material

Supplementary Material

## Figures and Tables

**Table 1:** Clinicopathological factors of *RSPO* fusion-positive and -negative colorectal cancer (N = 1019)

	Positive (n = 29)	Negative (n = 990)	P value
Age			0.82
Median	64	64	
Range	36-86	17-92	
Sex			0.036
Female	17 (59%)	389 (39%)	
Male	12 (41%)	601 (60%)	
Location			0.044^a^
Right colon	13 (45%)	275 (28%)	
Left colon	8 (28%)	418 (42%)	
Rectum	8 (28%)	297 (30%)	
Tumor grade			0.12
Low	26 (90%)	948 (96%)	
High	3 (10%)	42 (4%)	
Mucinous component			8.1 × 10^−7^
< 10%	16 (55%)	863 (87%)	
≥ 10%	13 (45%)	127 (13%)	
T stage			0.88^b^
1	0 (0%)	24 (2%)	
2	4 (14%)	103 (10%)	
3	18 (62%)	698 (71%)	
4	7 (24%)	165 (17%)	
N stage			0.53
0	13 (45%)	503 (51%)	
1–2	16 (55%)	487 (49%)	
M stage			0.96
0	23 (79%)	789 (80%)	
1	6 (21%)	201 (20%)	
Stage (UICC 8th ed)			0.48^c^
I	3 (10%)	96 (10%)	
II	9 (31%)	379 (38%)	
III	11 (38%)	314 (32%)	
IV	6 (21%)	201 (20%)	
MMR status^d^			0.33
Proficient	25 (86%)	900 (91%)	
Deficient	4 (14%)	85 (9%)	

a Right colon vs. Left colon + Rectum.

b 1+2 vs. 3+4.

c I+II vs. III+IV.

d Five tumors were excluded because of the unavailability of tissue blocks.

**Table 2:** Univariate and multivariate analyses of predictors associated with *RSPO* fusions

	Reference	Univariate analysis			Multivariate analysis		
		OR	95% CI	P value	OR	95% CI	P value
Age (≥ 65 years)	< 65 years	1.0	0.48–2.1	1.0			
Sex (Female)	Male	2.2	1.0–4.5	0.041	2.2	1.0–4.8	0.040
Location (Right side)	Left side	2.1	1.0–4.4	0.044			
Tumor grade (High)	Low	0.34	0.11–1.1	0.070	0.50	0.14–1.8	0.29
Mucinous component (Present)	Absent	5.5	2.6–12	6.6 × 10^−6^	5.3	2.5–11	2.0 × 10^−5^
MMR status (Deficient)	Proficient	1.9	0.66–5.2	0.24			
Stage (III + IV)	I + II	1.3	0.62–2.7	0.50			

OR, odds ratio; CI, confidence interval.

**Table 3:** Comparison of molecular features of *RSPO* fusion-positive colorectal cancer among several cohorts

	*RSPO* fusion-positive						*RSPO* fusion-negative				
	Present study (n = 29)	TCGA (n = 11)	S:CORT-A (n = 4)	S:CORT-B (n = 4)	Genentech (n = 7)	Total (n = 55)	TCGA (n = 568)	S:CORT-A (n = 344)	S:CORT-B (n = 176)	Genentech (n = 68)	Total (n = 1156)
MMR-D/MSI-H	4 (14%)	3 (27%)	0	0	0	7 (13%)	75 (13%)	15 (4%)	3 (2%)	15 (22%)	105 (9%)
*APC*	0	0	0	1 (25%)	0	1 (2%)	439 (78%)^b^	280 (81%)	160 (89%)	31 (46%)	750 (79%)^b^
*RNF43*	0	0	0	0	0	0	47 (8%)^c^	16 (5%)	2 (1%)	3 (4%)	66 (6%)^c^
*CTNNB1*	0	0	0	0	0	0	11 (2%)	2 (1%)	0	2 (3%)	15 (1%)
*KRAS*	19 (66%)	4 (36%)	1 (25%)	4 (100%)	5 (71%)	33 (60%)	233 (41%)	173 (50%)	92 (51%)	31 (46%)	437 (46%)
*BRAF*	7 (24%)	4 (36%)	3 (75%)	0	2 (29%)	15 (27%)	25 (4%)^d^	48 (14%)	6 (3%)	2 (3%)	75 (7%)^d^
*NRAS*	1 (3%)	1 (9%)	0	0	0	2 (4%)	35 (6%)	20 (6%)	9 (5%)	2 (3%)	66 (6%)
*TP53*	16 (55%)	4 (40%)^a^	2 (50%)	3 (75%)	5 (71%)	30 (55%)^a^	254 (46%)^e^	207 (60%)	121 (67%)	24 (35%)	485 (62%)^e^
*PIK3CA*	2 (7%)	3 (27%)	1 (25%)	1 (25%)	3 (43%)	10 (18%)	108 (19%)	72 (21%)	22 (12%)	15 (22%)	195 (19%)
*SMAD4*	3 (10%)	1 (9%)	1 (25%)	0	3 (43%)	8 (15%)	21 (4%)^f^	29 (8%)	8 (4%)	4 (6%)	54 (5%)^f^

## Data Availability

All data and materials generated and/or analyzed during the current study are available from the corresponding author upon reasonable request.
