# Supplementary material for: Clinicopathological and molecular characteristics of RSPO fusion-positive colorectal cancer
Source: Br J Cancer. Author manuscript; Available in PMC 2024 Dec 6. (PMC9470590; doi:10.1038/s41416-022-01880-w)
Supplement: Supplementary Material [file EMS181815-supplement-supplementary_Material.pdf]

ADDITIONAL INFORMATION

- **Supplementary Tables 1–5**
- **Supplementary Figures 1, 2**

Supplementary Table 1. Primers used in the multiplex PCR assay

Primer	Sequence
EIF3E exon 1 F	GCATCGCGCACTTTTTGGAT
PTPRK exon 1 F	ACTCGGCATGGATACGACTG
PTPRK exon 6 F	AGAAGTGACAAAAACTGACCAGG
PTPRK exon 7 F	ACCGAATATGAGATCCGAGTTC
PTPRK exon 13 F	TCCGCGCAAAGGATACAACA
RSPO2 exon 2 R	GAGGGAGACTCGCCACTCA
RSPO3 exon 2 R	CTCCTTGGCAGCCTTGACTA

Supplementary Table 2. Clinicopathological and molecular features of *RSPO* fusion-positive colorectal cancer

No.	Age	Sex	Location	Histology	Degree of differentiation	Mucinous component[a]	MAPK pathway gene mutation	*RSPO* fusion	MMR status	TNM (Stage)	Recurrence	Follow-up (months)
1	49	F	Rectum	NOS	Moderate	+	*KRAS* G12D	*EIF3E-RSPO2*	pMMR	T3N2M0 (III)	Yes	DOD (22)
2	75	F	Rectum	NOS	Moderate	−	*KRAS* G12D	*PTPRK* (e7)-*RSPO3*	pMMR	T3N1M0 (III)	No	NED (108)
3	70	M	Transverse	NOS	Poor	−	*BRAF* V600E	*PTPRK* (e1)-*RSPO3*	pMMR	T4N1M0 (III)	Yes	DOD (5)
4	55	F	Rectum	NOS	Poor	−	–	*PTPRK* (e13)-*RSPO3*	pMMR	T3N1M1 (IV)	Yes	DOD (14)
5	75	F	Ascending	NOS	Moderate	+	*KRAS* G12V	*PTPRK* (e1)-*RSPO3*	pMMR	T3N1M0 (III)	No	NED (97)
6	73	F	Ascending	NOS	Well	−	*KRAS* G12A	*PTPRK* (e1)-*RSPO3*	pMMR	T3N1M0 (III)	No	NED (96)
7	57	M	Ascending	Mucinous	Well	++	*KRAS* G13D	*PTPRK* (e1)-*RSPO3*	pMMR	T4N1M1 (IV)	–[b]	DOD (36)
8	57	F	Rectum	NOS	Well	+	*KRAS* G12D, *KRAS* Q61L	*PTPRK* (e1)-*RSPO3*	dMMR	T3N0M0 (III)	No	NED (116)
9	65	F	Transverse	NOS	Moderate	−	*BRAF* V600E	*PTPRK* (e1)-*RSPO3*	pMMR	T4N0M1 (IV)	Yes	DOD (36)
10	42	M	Rectum	NOS	Well	+	*BRAF* V600E	*EIF3E-RSPO2*	pMMR	T2N0M0 (I)	No	NED (78)
11	64	M	Cecum	Medullary	Poor	−	*BRAF* V600E	*EIF3E-RSPO2*	dMMR	T3N1M0 (III)	No	NED (96)
12	73	F	Rectum	NOS	Well	+	*KRAS* G12D	*PTPRK* (e1)-*RSPO3*	pMMR	T2N0M0 (I)	No	NED (92)
13	64	M	Rectum	NOS	Well	−	*KRAS* G12V	*PTPRK* (e7)-*RSPO3*	pMMR	T3N0M0 (II)	Yes	AWD (67)
14	54	F	Transverse	NOS	Moderate	−	*KRAS* G12D	*PTPRK* (e1)-*RSPO3*	pMMR	T3N1M0 (III)	No	NED (112)
15	61	M	Sigmoid	Mucinous	Well	++	*KRAS* G12D	*PTPRK* (e7)-*RSPO3*	pMMR	T4N1M1 (IV)	–[b]	DOD (25)
16	49	F	Sigmoid	Mucinous	Well	++	*BRAF* V600E	*PTPRK* (e7)-*RSPO3*	pMMR	T3N1M0 (III)	No	NED (43)
17	75	M	Transverse	NOS	Moderate	−	*NRAS* G13R	*PTPRK* (e1)-*RSPO3*	pMMR	T3N1M0 (III)	No	NED (63)
18	52	F	Ascending	NOS	Moderate	+	*KRAS* G12D	*PTPRK* (e1)-*RSPO3*	pMMR	T4N0M0 (II)	Yes	DOD (15)
19	68	F	Sigmoid	NOS	Well	+	*KRAS* Q61H	*PTPRK* (e13)-*RSPO3*	pMMR	T3N0M0 (II)	No	NED (56)
20	77	F	Ascending	NOS	Well	+	*KRAS* G12D	*PTPRK* (e1)-*RSPO3*	pMMR	T3N0M0 (II)	No	NED (49)
21	86	F	Ascending	NOS	Moderate	−	*KRAS* G12D	*PTPRK* (e1)-*RSPO3*	pMMR	T4N0M0 (II)	No	NED (41)
22	72	M	Sigmoid	NOS	Moderate	−	*KRAS* G12V	*EIF3E-RSPO2*	dMMR	T3N0M0 (II)	No	NED (46)
23	40	F	Sigmoid	NOS	Moderate	−	–	*PTPRK* (e1)-*RSPO3*	pMMR	T2N2M0 (III)	No	NED (43)
24	71	M	Ascending	NOS	Well	+	*BRAF* V600E	*PTPRK* (e1)-*RSPO3*	dMMR	T3N0M0 (II)	No	NED (43)
25	61	M	Sigmoid	Mucinous	Well	++	*BRAF* V600E	*EIF3E-RSPO2*	pMMR	T3N2M1 (IV)	No	NED (41)
26	68	M	Ascending	NOS	Moderate	−	*KRAS* G12C	*PTPRK* (e1)-*RSPO3*	pMMR	T3N1M0 (III)	Yes	AWD (42)
27	57	F	Sigmoid	NOS	Well	−	*KRAS* G12D	*PTPRK* (e1)-*RSPO3*	pMMR	T2N0M0 (I)	No	NED (15)
28	36	M	Sigmoid	NOS	Moderate	−	*KRAS* G12D	*PTPRK* (e1)-*RSPO3*	pMMR	T4N1M1 (IV)	No	NED (5)
29	78	F	Rectum	NOS	Moderate	−	*KRAS* G12D	*PTPRK* (e1)-*RSPO3*	pMMR	T3N0M0 (II)	No	NED (1)

F, female; M, male; NOS, adenocarcinoma, not otherwise specified; pMMR, mismatch repair-proficient; dMMR, mismatch repair-deficient; DOD, died of disease; NED, no evidence of disease; AWD, alive with disease

a Extent of mucin production: −, <10%; +, 10–50%; ++, > 50%.

b Local resection only.

Supplementary Table 3. Histological features of *RSPO* fusion-positive colorectal cancer

Findings	n
Histology	
Adenocarcinoma, NOS	24 (83%)
Mucinous adenocarcinoma	4 (14%)
Medullary carcinoma	1 (3%)
Lymphatic invasion	
Present	14 (48%)
Absent	15 (52%)
Venous invasion	
Present	15 (52%)
Absent	14 (48%)
Perineural invasion	
Present	11 (38%)
Absent	18 (62%)
Mucinous component	
< 10%	16 (55%)
10–50%	9 (31%)
≥ 50%	4 (14%)
Tumor infiltrating lymphocytes	
High	8 (28%)
Low	21 (72%)
Crohn's-like reaction	
Present	12 (41%)
Absent	17 (59%)
Precursor component	
Present	2 (7%)
Absent	27 (93%)

NOS, not otherwise specified

Supplementary Table 4. Comparison of clinicopathological features of *RSPO* fusion-positive colorectal cancer among several cohorts.

	RSPO fusion-positive					*RSPO* fusion-negative	P value
	Present study (n = 29)	TCGA (n = 11)	S:CORT-A (n = 4)	S:CORT-B (n = 4)	Total (n = 48)	(n = 2115)	
Age							0.91
Median	64	72	60	70	65	65	
Range	36-86	45–82	52–78	62–79	36–86	17–92	
Sex							0.033
Female	17 (59%)	6 (55%)	1 (25%)	3 (75%)	27 (56%)	865 (41%)	
Male	12 (41%)	5 (45%)	3 (75%)	1 (25%)	21 (44%)	1250 (59%)	
Location[b]		5	31				0.10[a]
Right colon	13 (45%)	6 (55%)	2 (50%)	0	21 (44%)	679 (32%)	
Left colon	8 (28%)	4 (36%)	2 (50%)	0	14 (29%)	799 (38%)	
Rectum	8 (28%)	1 (9%)	0	4 (100%)	13 (27%)	603 (29%)	
Mucinous component[b]			226				6.9×10^{-8}
< 10%	16 (55%)	7 (64%)	2 (50%)	NA[c]	25 (57%)	1475 (86%)	
≥ 10%	13 (45%)	4 (36%)	2 (50%)	NA[c]	19 (43%)	240 (14%)	
Stage[b]		16	11				
I	3 (10%)	0	0	NA[c]	3 (7%)	205 (11%)	
II	9 (31%)	6 (55%)	1 (25%)	NA[c]	16 (36%)	636 (33%)	
III	11 (38%)	5 (45%)	1 (25%)	NA[c]	17 (39%)	562 (29%)	
IV	6 (21%)	0	2 (50%)	NA[c]	8 (18%)	511 (27%)	

a Right colon vs. Left colon + Rectum.
b Location, mucinous component, stage data are missing for 36, 226, 27 cases, respectively, in *RSPO* fusion-negative group.
c Not assessed since all patients included in S:CORT-B cohort received neoadjuvant chemotherapy.

Supplementary Table 5. Univariate and multivariate analyses of overall and recurrence-free survivals in patients with stage II and III colorectal cancer

		Overall survival						Recurrence-free survival					
		Univariate analysis			Multivariate analysis			Univariate analysis			Multivariate analysis		
	Reference	HR	95% CI	P value	HR	95% CI	P value	HR	95% CI	P value	HR	95% CI	P value
RSPO fusion (Positive)	Negative	1.3	0.40–4.0	0.69	1.2	0.39–4.0	0.72	1.5	0.63–3.8	0.34	2.3	0.92–5.9	0.073
Age (≥ 65 years)	< 65 years	2.0	1.3–3.0	1.2×10^{-3}	2.0	1.3–3.0	1.5×10^{-3}	0.88	0.61–1.3	0.51	0.92	0.63–1.3	0.66
Sex (Female)	Male	0.62	0.41–0.96	0.032	0.59	0.38–0.92	0.020	0.52	0.34–0.79	2.1×10^{-3}	0.53	0.34–0.81	3.5×10^{-3}
Location (Right side)	Left side	1.0	0.65–1.6	0.96	1.3	0.79–2.0	0.32	0.41	0.25–0.69	8.1×10^{-4}	0.53	0.31–0.91	0.020
MMR status (Deficient)	Proficient	0.36	0.15–0.88	0.025	0.41	0.16–1.1	0.066	0.20	0.063–0.63	$5.9 x 10^{-3}$	0.33	0.10–1.1	0.067
Stage (III)	II	1.8	1.2–2.7	3.5×10^{-3}	1.7	1.2–2.6	8.0×10^{-3}	2.5	1.7–3.7	5.7×10^{-6}	2.5	1.6–3.7	1.1×10^{-5}

HR, hazard ratio; CI, confidence interval.

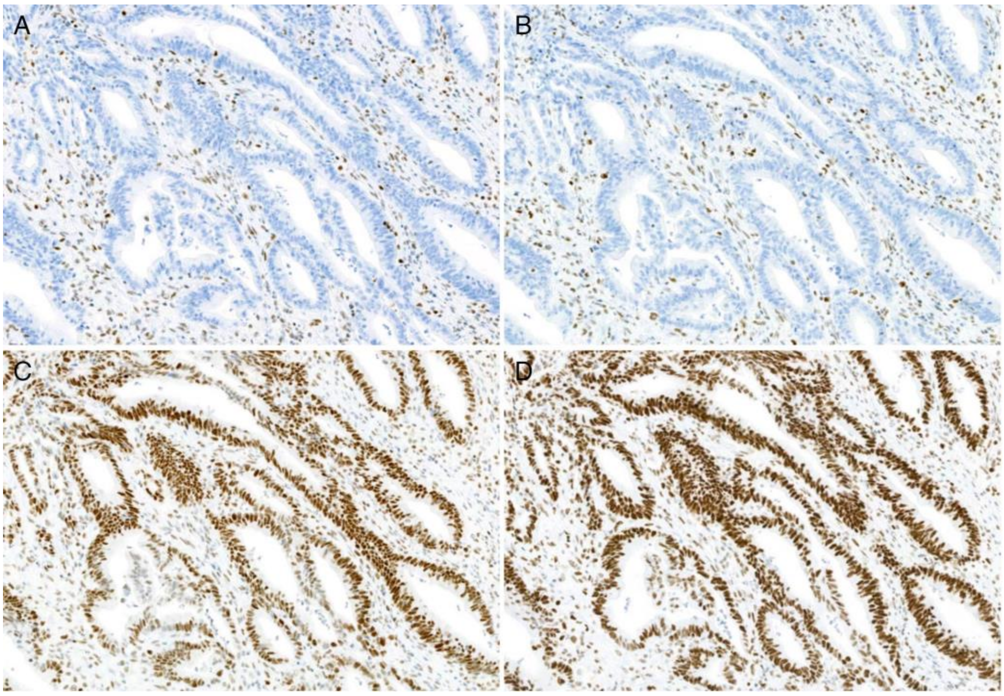

Supplementary Figure 1. Expression of mismatch repair proteins in *RSPO* fusion-positive colorectal cancer.

A–D: Loss of mismatch repair protein expression in an adenocarcinoma with an *RSPO* fusion. Tumor cells showed concurrent loss of MLH1 (A) and PMS2 (B), with a retained expression of MSH2 (C) and MSH6 (D).

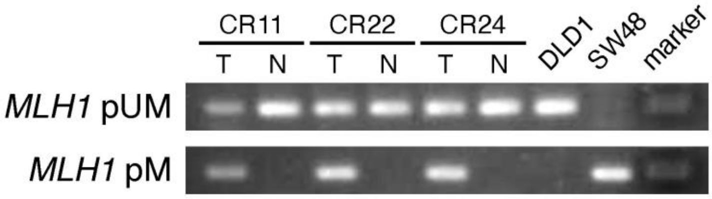

Supplementary Figure 2. Analysis of the *MLH1* promoter methylation status. pUM and pM indicate the presence of unmethylated and methylated alleles, respectively. DNA samples from DLD1 and SW48 cell lines were used as methylated and unmethylated controls, respectively. T, Tumor; N, Normal mucosa.
